# Assessing volume responsiveness using right ventricular dynamic indicators of preload

**DOI:** 10.1007/s00540-021-02937-5

**Published:** 2021-05-05

**Authors:** Michael F. Graessler, Karin H. Wodack, Hans O. Pinnschmidt, Sarah Nishimoto, Christoph R. Behem, Daniel A. Reuter, Constantin J. C. Trepte

**Affiliations:** 1 Department of Anesthesiology, Centre for Anesthesiology and Intensive Care Medicine, University Medical Center Hamburg-Eppendorf, Martinistr. 52, 20246 Hamburg, Germany; 2 Department of Medical Biometry and Epidemiology, University Medical Center Hamburg-Eppendorf, Hamburg, Germany; 3 Department of Anesthesiology and Intensive Care Medicine, University Medical Center Rostock, Rostock, Germany

**Keywords:** Right ventricle, Volume responsiveness, Dynamic indicators, Pulmonary artery catheter

## Abstract

**Purpose:**

Dynamic indicators of preload currently only do reflect preload requirements of the left ventricle. To date, no dynamic indicators of right ventricular preload have been established. The aim of this study was to calculate dynamic indicators of right ventricular preload and assess their ability to predict ventricular volume responsiveness.

**Materials and methods:**

The study was designed as experimental trial in 20 anaesthetized pigs. Micro-tip catheters and ultrasonic flow probes were used as experimental reference to enable measurement of right ventricular stroke volume and pulse pressure. Hypovolemia was induced (withdrawal of blood 20 ml/kg) and thereafter three volume-loading steps were performed. ROC analysis was performed to assess the ability of dynamic right ventricular parameters to predict volume response.

**Results:**

ROC analysis revealed an area under the curve (AUC) of 0.82 (CI 95% 0.73–0.89; *p* < 0.001) for right ventricular stroke volume variation (SVV_RV_), an AUC of 0.72 (CI 95% 0.53–0.85; *p* = 0.02) for pulmonary artery pulse pressure variation (PPV_PA_) and an AUC of 0.66 (CI 95% 0.51–0.79; *p* = 0.04) for pulmonary artery systolic pressure variation (SPV_PA_).

**Conclusions:**

In our experimental animal setting, calculating dynamic indicators of right ventricular preload is possible and appears promising in predicting volume responsiveness.

## Introduction

The assessment and prediction of fluid responsiveness are key elements for the management of fluid therapy. In clinical practice, dynamic left ventricular indicators of preload, such as stroke volume variation (SVV) or pulse pressure variation (PPV), have been shown to be the most suitable parameters to detect and predict volume responsiveness [1–3].

To date, dynamic indicators of preload have only been studied for systemic circulation and in this context, only provide information on the preload requirements of the left ventricle. An important aspect that is often forgotten in relation to SVV and PPV is that not only controlled ventilation and sinus rhythm, but also normal right ventricular function are indispensable prerequisites [4]. Knowledge of right ventricular preload and preload requirements is crucial since, due to its lower contractile reserve, the right ventricle is particularly sensitive to modifications of preload or afterload [5]. Therefore, the right heart represents the weakest link in the chain when it comes to assessing preload requirements as well as volume responsiveness. As a result, in clinical situations where due to volume overload cardiac deterioration occurs, right ventricular deterioration is much more likely to occur first [6]. Right ventricular impairment may actually aggravate this situation, making the right ventricle even more dependent on adequate preload and more vulnerable to change. Acute pulmonary artery embolism, cor pulmonale in acute respiratory failure or right ventricular myocardial infarction are typical causes of right ventricular impairment in critically ill patients.

One reason why estimating right ventricular preload demand still presents enormous difficulties is that right ventricular preload variables, and fluid responsiveness in particular, are much more difficult to assess methodologically. The pressure-based parameter, central venous pressure (CVP), may be associated with right ventricular preload to some extent, but numerous studies have shown that CVP fails to predict fluid responsiveness properly [7–9]. Right ventricular volumetric parameters of preload, such as right ventricular end-diastolic volume (RVEDV), were also found to be poor predictors [10]. In right ventricular dysfunction, left ventricular SVV is typically high because of relative left ventricular hypovolemia due to right ventricular dysfunction. In such situations, there is actually no suitable parameter to predict the fluid responsiveness. However, previous publications have shown that during controlled mechanical ventilation there are also changes in right ventricular stroke volume due to changes in intrathoracic pressures and volumes [11–13]. This provides the possibility to calculate dynamic indicators of right ventricular preload as well.

To date, there is only one experimental study specifically aimed at quantifying right ventricular heart–lung interaction. In this study, right ventricular stroke volume variation (SVV_RV_) was recorded during controlled mechanical ventilation and evaluated in two different preload situations. The authors concluded that SVV_RV_ appears to reflect the preload demands of the right ventricle [14]. However, no data exist on whether these parameters are able to predict the right ventricular volume response.

Thus, identifying a parameter to predict right ventricular fluid responsiveness is of highest clinical interest, as there are many clinical situations in which the right ventricle is likely to be crucial in assessing a patient’s preload requirements [15–17]. The aim of this study was to calculate dynamic right ventricular preload parameters and evaluate their ability to properly predict fluid responsiveness in a controlled experimental model.

## Methods

### Ethical statement

Ethical approval for the trial (Ethical Committee N^o^ 53/11) was provided by the local Governmental Commission on the Care and Use of Animals, Hamburg, Germany (Chairperson Karolin Zoll, PhD) on August 04, 2011. The animals received care in compliance with the “Guide for the Care and Use of Laboratory Animals”. The project was carried out according to the ARRIVE Guidelines [18].

### Study design

The study was designed as a experimental trial in 20 domestic pigs.

### Experimental procedures

The experiments were conducted in a standardized environment in an animal operating theater. Animals were fasted overnight and anesthesia was induced with intramuscular ketamine 10 mg/kg, midazolam 0.3 mg/kg, azaperone 4 mg/kg and atropine 0.5 mg.

Anesthesia was maintained by continuous intravenous fentanyl (10 µg/kg/h) and inhaled sevoflurane (end-expiratory concentration 2.5%). Animals were mechanically ventilated with a tidal volume of 8 ml/kg bodyweight and a positive end-expiratory pressure of 5 cmH2O (Zeus®; Draeger Medical, Lübeck, Germany). The ventilation frequency was set to maintain an end-expiratory arterial carbon dioxide tension (pCO2) of 35–40 mmHg and was not changed throughout the experimental protocol.

A 7.5 Fr and a 12 Fr central venous catheter were inserted into the right internal and external jugular vein (Certofix® Trio S 730, 30 cm, Certofix® Trio HF S1220, 20 cm, B.Braun, Melsungen, Germany). An arterial line was placed in the right femoral artery. A continuous infusion rate of 10 ml/kg of a balanced solution (Sterofundin®, B.Braun, Melsungen, Germany) was applied. A sternotomy was performed and an ultrasound flow probe (Confidence PAU Flowprobe®, Chronic Liner, 16 mm, Transonic Systems Inc., Ithaca, NY, USA) was placed around the pulmonary artery and connected to a flowmeter (Perivascular Flow Module®, Transonic Systems Inc., Ithaca, NY, USA) to measure right ventricular stroke volume and calculate stroke volume variation using an experimental reference [19]. A pressure catheter was inserted into the pulmonary artery and fixed with a pouch suture (Millar Micro-Tip® pressure catheter, Houston, Texas, USA). A patch was then sewn into the pericardium, the sternum was re-approximated with cerclage wires and the soft tissues were closed in layers to restore closed chest conditions. During surgical preparation, meticulous care was taken not to injure the pleural cavities on either side. Warming blankets and heated infusions were used to keep the body temperature constantly above 37 °C.

### Experimental animals

The study was conducted on 20 German Landrace domestic pigs with a mean age of 3 months and a mean body weight of 36.7 ± 4.7 kg, 11 males and 9 females.

### Housing and husbandry

The animals arrived at the animal facilities of the University Medical Center Hamburg-Eppendorf 10 days before the experiments to allow acclimatization and recovery from transport-related stress. The animals were kept in an enriched environment.

### Experimental results and measurements

**Fig. 1:**
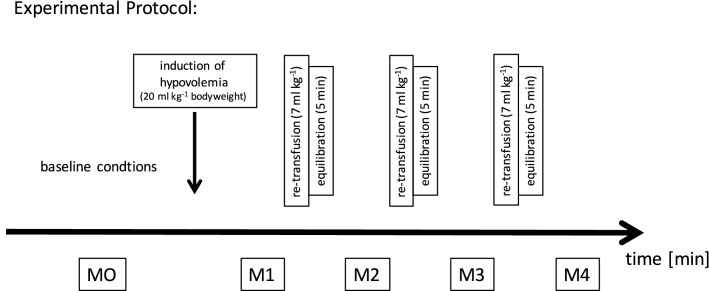
Schematic display of the experimental protocol containing the volume withdrawal and the re-transfusion steps

After baseline measurements, hypovolemia was induced. Within 15 min, 20 ml/kg body weight of blood was removed. The withdrawn blood was collected in a special blood bag (CompoFlex P 4170, FreseniusKabi, Bad Homburg, Germany). After 5 min for equilibration, measurements (M1) were performed. Re-transfusion of the drained blood was then initiated in three steps over 5 min each at 7 ml/kg body weight. After each step, 5 min was allowed for equilibration before measurements (M2–M4). A schema of the experimental procedure is shown in Fig. 1.

### Data collection and processing

Data were recorded at M0-M4 using adapted hardware from ADInstruments (ADInstrumentsPowerLab®, ADInstruments Ltd, Oxford, UK) and Transonic (Perivascular Flow Module, Transonic Systems Inc, Ithaca, NY, USA). Data analysis was performed offline using LabChart® software (LabChart Pro, version 8, ADInstruments, Oxford, United Kingdom).

Right ventricular stroke volume variation (SVV_RV_) was calculated offline from the pulmonary artery flow signal. Pulmonary artery pulse pressure variation (PPV_PA_) and systolic pressure variation (SPV_PA_) were calculated from pulmonary artery pressure tracings. Calculations of mean right ventricular stroke volume, pulse pressure and systolic pressure were performed on 10 respiratory cycle data using the following formulae:

$${\text{SVV}}_{{{\text{RV}}}} \left( \% \right) = \frac{{({\text{SVmax}}_{{{\text{RV}}}} {-}{\text{ SVmin}}_{{{\text{RV}}}} )}}{{({\text{SVmean}}_{{{\text{RV}}}} )}} \times 100$$

$${\text{PPV}}_{{{\text{PA}}}} \left( \% \right) = \frac{{({\text{PPmax}}_{{{\text{PA}}}} {-}{\text{ PPmin}}_{{{\text{PA}}}} )}}{{({\text{PPmean}}_{{{\text{PA}}}} )}} \times 100$$

$${\text{SPV}}_{{{\text{PA}}}} \left( \% \right) = \frac{{({\text{SPmax}}_{{{\text{PA}}}} {-}{\text{ SPmin}}_{{{\text{PA}}}} )}}{{({\text{SPmean}}_{{{\text{PA}}}} )}} \times 100$$

### Statistical methods

The ability to predict volume responsiveness was assessed for each variable by nonparametric estimation of the receiver-operating characteristic (ROC) curves and their AUCs [20]. Considering the three time points together as cluster data. ANOVA-type statistics were evaluated to examine whether an individual AUC was significantly different from 0.5. The response to volume application was considered positive, if right ventricular stroke volume increased by at least 15% (criterion value) [3, 19, 20]. Ideal cutoff values were identified by calculating the Youden Index [21]. Mixed models were fit to data of the dependent variables MAP, HR, RV_SV_, SVV_RV_, MPAP, SPV_PA_, PPV_PA_ and CVP assuming a fixed effect for point of measurement and random intercepts for animals. This was followed by post hoc comparisons of points of measurement with preceding points of measurement via pairwise contrasts. Model-estimated marginal means and their 95% confidence intervals are presented. Prior to mixed model analyses, histograms of data of the dependent variables were visually examined for normal distribution. Statistical analysis was performed using SPSS® for Windows® (IBM® SPSS Statistics version 25.0; Armonk, NY, USA) except for ROC analyses that were done using R. Statistical, significance was appraised for *p* < 0.05.

## Results

### Hemodynamic data

Induction of hypovolemia resulted in a significant decrease of mean arterial pressure (MAP), mean pulmonary artery pressure (MPAP) as well as CVP and a significant increase of dynamic parameters of preload of the right ventricle (SVV_RV_, PPV_PA_ and SPV_PA_) from M0 to M1. All hemodynamic data throughout the experimental protocol are presented in Table 1.

**Table 1:** Hemodynamic parameters throughout the experimental protocol

	Baseline	Hypovolemia	1st volume-loading step	2nd volume-loading step	3rd volume-loading step
HR (min^−1^)	103 [97;110]	101.845 [94;107]	104 [97;110]	101[95;108]	104 [97;110]
MAP (mmHg)	78 [74;82.6]	62 [57;66] *	73 [68;77]*	73 [69;77]	79 [76;84]*
mPAP (mmHg)	35 [33;37]	28 [26;31]*	32 [29;34]*	32 [29; 34]	33 [30;35]
SV_RV_ (ml)	29 [27;31]	21 [19;23]*	25 [23;27]*	26 [24;28]	27 [24;28]
SVV_RV_ (%)	35,680 [28;39]	55 [49;60]*	42,614 [37;48]*	40 [35;45]	35,960 [31;41]*
PPV_PA_ (%)	47 [40;49]	60 [53;67] *	56 [49;63]*	50 [43;56]*	51 [44;57,652]
SPV_PA_ (%)	18 [15;21]	23 [20;26] *	22 [19;25]	20 [18; 23]	20 [17; 23]
CVP (mmHg)	5.9 [4.2;7.5]	3.9 [2.2; 5.6] *	4.9 [3.2; 6.6]*	6.1 [4.4; 7.7]*	5.9 [4.3; 7.6]

Data are presented as mixed model-estimated marginal means with 95% confidence intervals. Model-estimated marginal means and their 95% confidence intervals are presented. Prior to mixed model analyses, histograms of data of the dependent variables were visually examined for normal distribution

*HR* heart rate, *MAP* mean arterial pressure, *mPAP* mean pulmonary artery pressure, *SV*_*RV*_ right ventricular stroke volume, *SVV*_*RV*_ right ventricular stroke volume variation, *PPV*_*PA*_ pulmonary artery pulse pressure variation, *SPV*_*PA*_ pulmonary artery systolic pressure variation, *CVP* central venous pressure

*Significantly different from preceding point of measurement (*p* < 0.05)

### Prediction of volume responsiveness

**Fig. 2:**
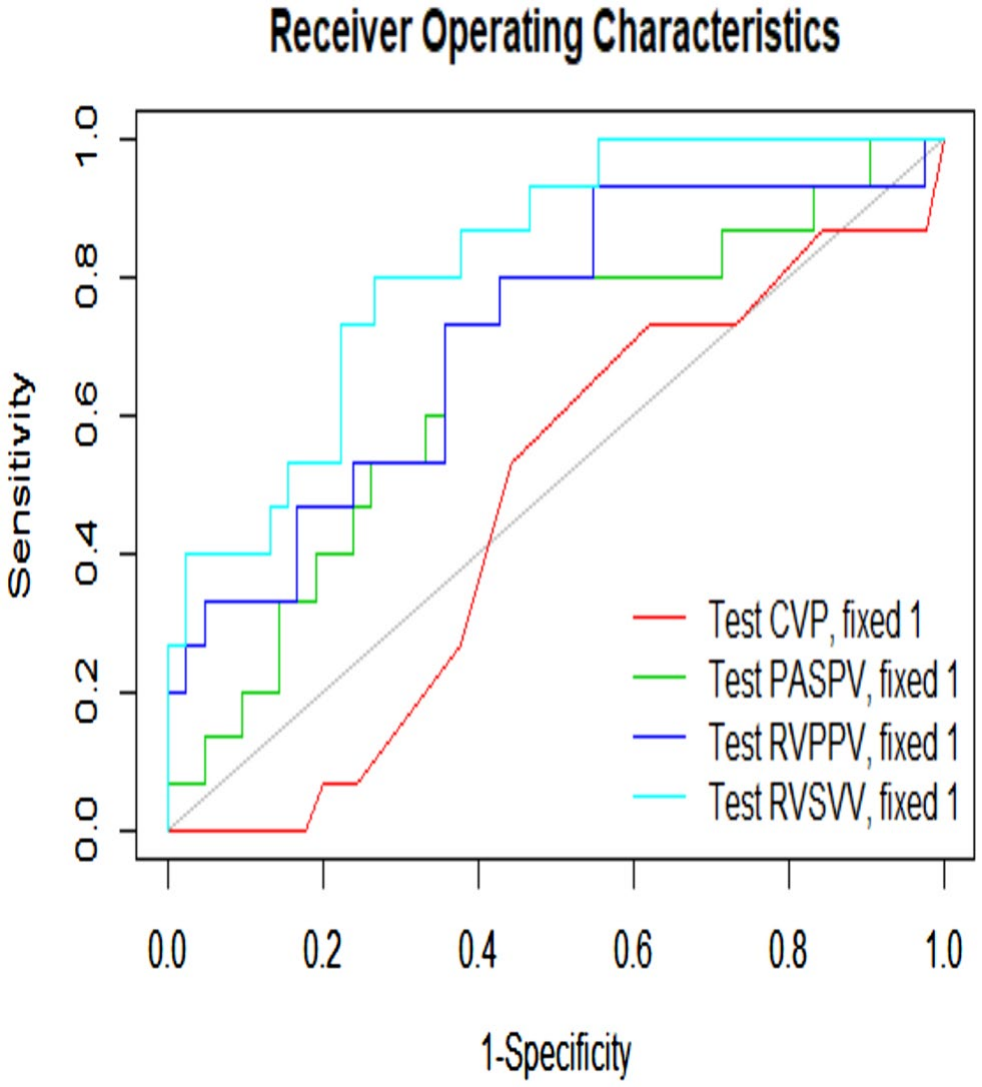
Receiver-operating characteristic (ROC) for right ventricular stroke volume variation (SVV_RV_), pulmonary artery pulse pressure variation (PPV_PA_), pulmonary artery systolic pressure variation (SPV_PA_) and central venous pressure (CVP) with reference line (light blue). Areas under the curves (AUC) are given beside the curves

In each animal, 3 volume-loading steps were performed, thus in total 60 volume-loading steps were analyzed except for SPV and PPV that had only 57 valid observations due to missing data. The ROC curves are presented in Fig. 2. In detail SVV_RV_ presented with an AUC of 0.82 (CI 95% 0.73–0.89; *p* < 0.001), PPV_PA_ with an AUC of 0.72 (CI 95% 0.53–0.85; *p* = 0.02) and SPV_PA_ with an AUC of 0.66 (CI 95% 0.51–0.79; *p* = 0.04). CVP presented with an AUC of 0.47 (CI 95% 0.33–0.62; *p* = 0.71).

According to the ROC analysis in this experimental model in pigs, the following cutoff values for the prediction of volume responsiveness can be proposed: SVV_RV_ > 45.8% (Youden Index 0.53; sensitivity 80%; specificity 73.3%), PPV_PA_ > 46.2% (Youden Index 0.39; sensitivity 93.3%; specificity 45.2%), SPV_PA_ > 21.1% (Youden Index 0.36; sensitivity 73.3%; specificity 63.1%) and CVP < 3 (Youden Index 0.11; sensitivity 73.3%; specificity 37.8%).

## Discussion

Our study provides the first results regarding the possibility of predicting the volume response by calculating SVV_RV_, PPV_PA_ and SPV_PA_ as dynamic right ventricular indicators of preload in an experimental animal model. The data show that SVV_RV_ in particular has a valuable predictive capability and may has the potential to assess volume response and guide volume therapy in the future [10].

Although right ventricular heart–lung interactions have been described in the literature [22, 23], surprisingly, no attempt has been made to calculate and assess the ability to predict volume responsiveness using right ventricular dynamic indicators of preload. This may be due to the fact that hemodynamic variables, used to assess preload and predict volume responsiveness derived from the right ventricle, are much more difficult to assess methodologically. Our results demonstrate for the first time the ability of dynamic parameters to predict fluid response based on hemodynamic signals from the right ventricle and pulmonary circulation. Pulmonary artery pulse pressure and systolic pressure can be derived from a pulmonary artery catheter, however, there is no system that allows online measurement and calculation of PPV_PA_ and SPV_PA_ in a clinical application. The only previous study reporting the calculation of right ventricular SVV as a dynamic indicator was published by Kubitz et al. The authors calculated SVV_RV_ in hypovolemia and during two other loading conditions in 15 anesthetized and ventilated pigs. They were able to show that a significant increase in SVV_RV_ occurred during volume depletion and a consequent decrease with re-transfusion. It was concluded that SVV_RV_ seemed to reflect right ventricular volume requirements [14]. However, no evaluation of SVV_RV_ was performed to predict volume response.

An indispensable prerequisite for dynamic preload indicators is that they require not only controlled mechanical ventilation and sinus rhythm, but also adequate right ventricular function to properly predict fluid responsiveness. If right ventricular function is impaired and right ventricular stroke volume decreases, reduced left ventricular preload and relative hypovolemia result. Under these conditions, left ventricular dynamic indicators of preload cannot differentiate absolute hypovolemia from relative left ventricular hypovolemia due to right ventricular impairment. These aspects highlight the importance of identifying new predictors of volume responsiveness based on right ventricular hemodynamics. In clinical situations where cardiac deterioration occurs due to volume overload, it is much more likely that right ventricular decline will occur first due to smaller muscle mass and lower contractile reserve. Therefore, sometimes monitoring volume responsiveness using dynamic right ventricular preload indicators could be a very attractive alternative for high-risk patients.

In a multicenter study of patients undergoing coronary artery revascularization, Ranucci et al. showed that in the presence of poor right ventricular function, no suitable parameter for predicting fluid responsiveness could be identified [16]. In another experimental study, it was found that induction of experimental right ventricular failure by increasing MPAP resulted in a significant increase in left ventricular PPV and SVV, whereas they remained unchanged during the subsequent volume challenge and failed to predict volume responsiveness [25]. In the presence of increased pulmonary artery pressures or right ventricular dysfunction, established left ventricular dynamic indicators of preload also fail to correctly predict fluid responsiveness [24, 25]. Dynamic indicators of preload derived from right ventricular and pulmonary artery hemodynamic signals could help finding an individualized optimal position on the Frank–Starling curve and optimizing preload according to the precarious needs of the right ventricle. In addition, they may have the potential to distinguish hypovolemia from right ventricular impairment. This could be of particular interest when right ventricular impairment is already present and very precise volume therapy is needed, as volume overload can be even more deleterious and lead to further deterioration of right ventricular function [23, 26].

SVV_RV_, PPV_PA_ and SPV_PA_, reflecting right ventricular preload conditions, showed different and higher cutoff values compared to dynamic indicators of preload derived from the left ventricle. The higher cutoff values are coherent with the reported cutoff values of about 40% for the Superior Vena Cava Collapsibility Index, which is calculated as the difference in the diameter of the vena cava during the respiratory cycle [24]. These cutoff values reflect the high compliance of the venous system with consecutive changes in right ventricular preload, which are also reflected in SVV_RV_ and PPV_PA_. Another possible explanation for the much higher cutoff values for SVV_RV_ and PPV_PA_ in our study, compared with those reported for left ventricular SVV and PPV, could be the high pulmonary vascular compliance. The pulmonary vasculature and capillary bed act as a kind of filter, smoothing the effects before they reach the left heart, with consecutive lower values for left ventricular SVV and PPV. In addition, the right ventricle, with its lower contractile reserve, has a higher preload dependence. Due to these aspects, even small changes in right ventricular preload lead to relevant changes in right ventricular stroke volume and higher SVV_RV_ compared to left ventricular SVV.

At this time, we cannot present data that precisely quantify to what extent variations in venous return or changes in right ventricular afterload contribute to the occurrence of SVV_RV_ and PPV_PA_, but the literature reports that afterload appears to be of minor importance [25]. This is an important aspect that should be investigated in future studies. A comparison of AUCs in our study shows the highest value for SVV_RV_. One possible explanation is that SVV is less affected by changes in arterial vasomotor tone compared with PPV. An effect that has already been reported for left ventricular dynamic indicators of preload [27, 28]. Although CVP is a parameter that shows an association with right ventricular preload, in our results, with an AUC of 0.47, CVP is no better than a fifty–fifty chance and fails to predict right ventricular volume response, as shown in previous studies [7, 8, 29].

In fact, there are some limitations of our study. One aspect that needs to be considered is that our studied animals had relatively high baseline pulmonary artery pressures. Our understanding is that this was due to our experimental setup and surgical preparation. Since we used a micro-tip catheter in the pulmonary artery to achieve the best possible signal quality, a pouch suture on the pulmonary artery was required. This inevitably led to some reduction in vessel diameter and consequently an increase in pulmonary artery resistance and pressure. Another aspect that may have had some influence was the use of ketamine as an anesthetic, as ketamine increases systemic and pulmonary vascular resistance. However, only a single dose of ketamine was administered for induction of anesthesia and given the elimination half-life of ketamine, this effect should have been less important. The fact that dynamic right ventricular indicators of preload can only be derived using a pulmonary artery catheter limits their clinical applicability to a group of high-risk or critically ill patients. Since the primary focus of this study was to assess volume responsiveness based on right ventricular dynamic indicators of preload, no hemodynamic data from the systemic circulation were obtained in our experimental setup. Therefore, we cannot present data on the behavior of left ventricular dynamic indicators of preload (SVV_LV_, PPV, SV_LV_, CO) and compare them with our results.

Moreover, dynamic right ventricular indices of preload are likely to have the same methodological limitations as established left ventricular parameters. That is, they require controlled mechanical ventilation and regular cardiac sinus rhythm [30, 31]. Ventilator settings will also affect right ventricular SVV_RV_ and PPV_PA_. Since this is the first study to investigate their ability to predict volume response, we do not know whether these parameters require the same ventilator settings as left ventricular SVV and PPV. In our experimental setup, we chose the ventilator settings recommended for dynamic left ventricular indicators of preload. Differences in ventilator settings would also affect the cutoff values of these parameters, wherefore the reported cutoff values apply to our ventilator settings only. These aspects were not investigated in our study and should be addressed before these parameters can potentially be translated into clinical practice. So far, we can only postulate the advantage, that evaluation of right ventricular volume responsiveness may be of particular value to guide volume therapy in conditions of right ventricular failure, but we cannot present data in right ventricular impairment at this time.

In conclusion, the results of our study demonstrate the ability to predict volume responsiveness by calculating dynamic indicators of right ventricular preload in an experimental animal model. The results show that SVV_RV_ and PPV_PA_ in particular have reasonable predictive ability and have the potential to become valuable tools for assessing volume responsiveness and guiding fluid therapy in the future.

## Declarations

### Presentation

LIVES 2017 30th Annual Congress of the European Society of Intensive Care Medicine.
